# Microarray profiling reveals the integrated stress response is activated by halofuginone in mammary epithelial cells

**DOI:** 10.1186/1756-0500-4-381

**Published:** 2011-10-05

**Authors:** Yana G Kamberov, Jihoon Kim, Ralph Mazitschek, Winston P Kuo, Malcolm Whitman

**Affiliations:** 1 Genetics Department, Harvard Medical School, Boston, MA, USA; 2 Division of Biomedical Informatics, University of California, San Diego, CA, USA; 3 Center for Systems Biology, Chemical Biology Platform, Massachusetts General Hospital, Boston, MA and Infectious Diseases Initiative, The Broad Institute of Harvard University and MIT, Cambridge, MA, USA; 4 Laboratory for Innovative Translational Technologies, Harvard Medical School, Boston, MA, USA; 5 Department of Developmental Biology, Harvard School of Dental Medicine, Boston MA, USA

## Abstract

**Background:**

The small molecule Halofuginone (HF) is a potent regulator of extracellular matrix (ECM ) gene expression and is unique in its therapeutic potential. While the basis for HF effects is unknown, inhibition of TGFβ signaling and activation of the amino acid restriction response (AAR) have been linked to HF transcriptional control of a number of ECM components and amelioration of fibrosis and alleviation of autoimmune disease by regulation of Th17 cell differentiation, respectively. The aim of this study was to generate a global expression profile of HF targets in epithelial cells to identify potential mediators of HF function in this cell type.

**Results:**

We report that HF modulation of the expression of the ECM remodeling protein Mmp13 in epithelial cells is separable from previously reported effects of HF on TGFβ signal inhibition, and use microarray expression analysis to correlate this with transcriptional responses characteristic of the Integrated Stress Response (ISR).

**Conclusions:**

Our findings suggest activation of the ISR may be a common mechanism underlying HF biological effects.

## Background

**Figure 1:**
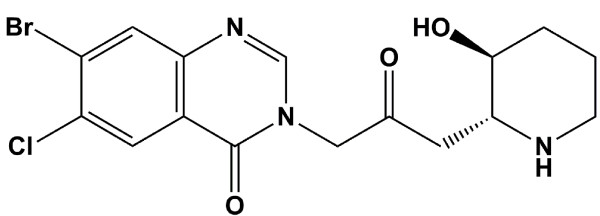
**Halofuginone (HF)**.

Natural product small molecules and their derivatives have had a profound impact on the development of treatments for a variety of human diseases. Understanding the molecular pathways mediating the biological effects of clinically useful small molecules facilitates the development better-tailored therapeutics. In recent years, considerable interest sparked around Halofuginone (HF) rel-7-bromo-6-chloro-3-[3-[(2R,3S)-3-hydroxy-2-piperidinyl]-2-oxopropyl]- 4(3H)-Quinazolinone, a racemic derivative of the anti-malarial plant quinazoline febrifugine {[3-[(2R,3S)-3-hydroxy-2-piperidinyl]-2-oxopropyl]-4(3H) quinazolinone} (Figure 1). Initially used as a broad-spectrum anti- protozoal, interest in the clinical potential of HF was spurred by the discovery that HF can ameliorate pathological extracellular matrix (ECM) deposition and remodeling [1,2]. These and numerous subsequent studies have established that HF is a potent anti-fibrotic, anti-angiogenic, anti-metastatic and ECM modulator *in vivo*, and an inhibitor of ECM gene expression, epithelial to mesenchymal (EMT) and fibroblast to myofibroblasts transition in vitro [3-9]. As a result, HF is in clinical trials for the treatment of scleroderma and as an anti-angiogenic for the treatment of Kaposi's sarcoma and solid tumors [10-13].

HF inhibition of Transforming Growth Factor-β (TGFβ) signaling has been proposed as a major mechanism for HF biological activity [8,14,15]. Treatment of cultured cells with HF has been linked to decreased levels of Phospho-Smad3, and specifically in epithelial cells, this has been proposed to result from HF-mediated transcriptional up-regulation of Smad7, a negative regulator of the pathway [14]. However, HF concentrations required to inhibit TGFβ signaling in epithelial cells are substantially higher (>100 nM) than concentrations shown to have biological consequences at the level of gene expression (1-10 nM) or HF plasma levels in animal models where HF exhibited biological activity (4 nM -10 nM) [2,4,5,14].

Recent work by Sundrud and colleagues, including our lab, (2009) demonstrates that HF modulates effector T-cell differentiation by activating the amino acid restriction response (AAR) in vivo and in vitro [16]. Importantly, AAR stress recapitulates the specificity of this modulation, indicating that AAR activation explains the effects of HF on T-cells [16]. This activity of HF occurred without any detectable change in TGFβ signaling in treated T-cells [16]. These data have been used to suggest that regulation of TGFβ signaling is not necessary for the biological effects of HF in T-cells. This raises the question if such a mechanism may also be employed in other cell types that may be targets for HF action, such as fibroblasts and epithelia. Because ECM remodeling, and epithelial-mesenchymal transition, may link changes in epithelial cell phenotype to fibrosis, we have investigated early responses to HF in this cell type [17-21].

NMuMG mammary epithelial cells have previously been shown to respond to HF at the level of Smad7 expression and Phospho-Smad3 down-regulation, albeit at the high dose of 100 nM HF [14]. In an effort to determine the early effectors mediating HF activity, we undertook an unbiased approach to examine early, sensitive responses to HF in this cell type. We show that treatment of cultured epithelial cells with HF results in upregulation of Mmp13, a reported HF target and that this response is clearly separable from Smad7 activation and inhibition of TGFβ signaling [22]. Using this assay as a backdrop, we performed genome wide microarray analysis and report the expression profile of HF treated cells. We find that HF treatment induces the transcription of many genes characteristic of an ATF4-mediated Integrated Stress Response (ISR) [16,23,24]. The HF transcriptional targets from this study provide a quantitative biological readout of the early downstream effects of HF, and suggest that HF activity in epithelial cells results in transcriptional activity characteristic of an ISR stress. Importantly, while the ISR can be triggered by several defined stressors, the upregulation of known AAR-specific markers, and absence of ER stress targets in our data, suggest that HF does not generally activate ER stress responses and is more consistent with a function for HF in the specific activation of the AAR that is shared between epithelial cells and T-cells [16,23,24]. Thus, our results point to AAR activation as a more general component of the biological response to HF.

## Results

### Modification of Halofuginone into transcriptionally active and inactive functional analogs

**Figure 2:**
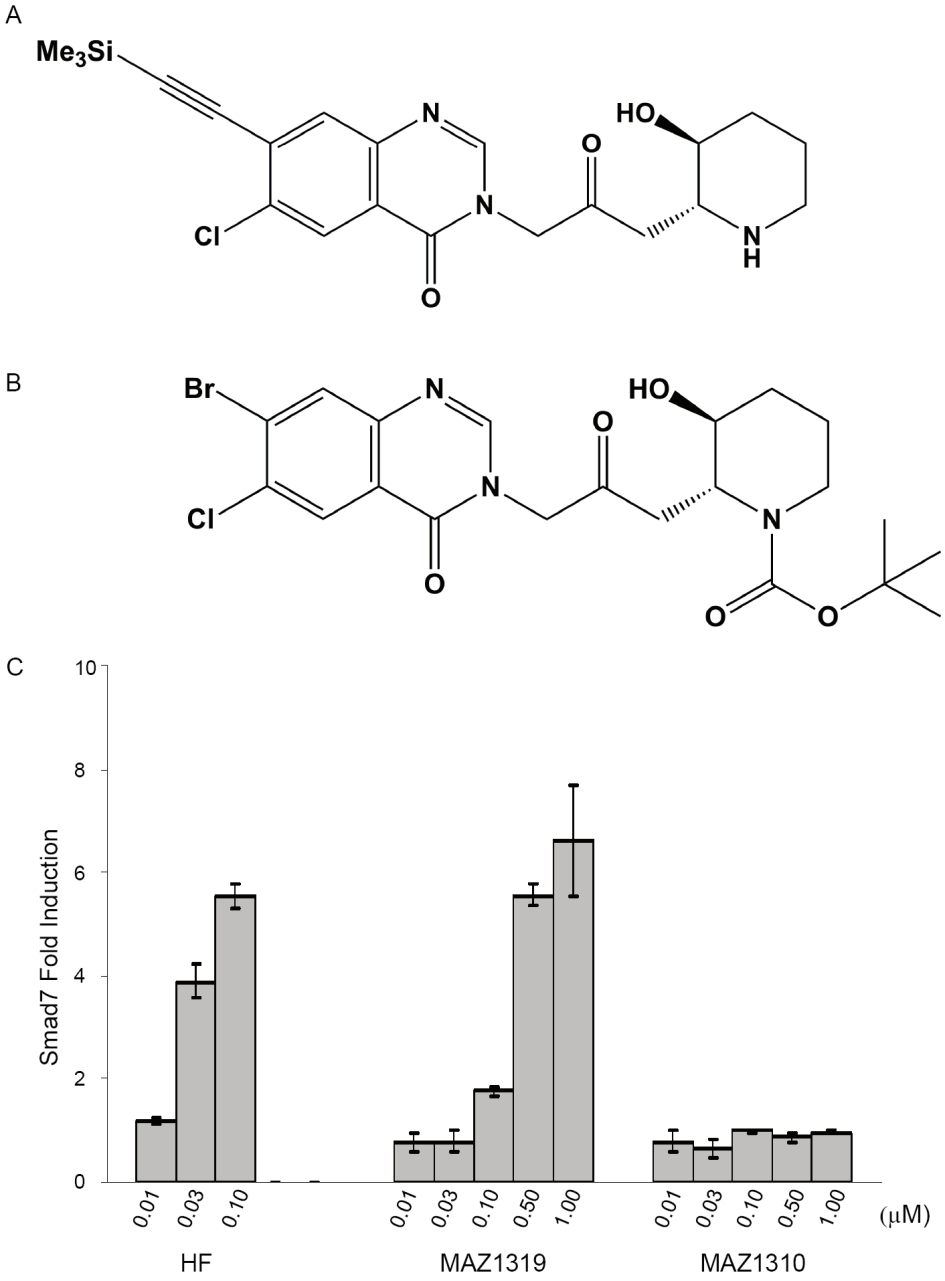
**HF can be modified into functional and non-functional analogs**. (A) Structures of MAZ1319 and (B) MAZ1310. (C) HF and MAZ1319 but not MAZ1310 induce transcription of Smad7 (C) in NMuMG cells after 8 hours of treatment. All samples were normalized to β-Actin transcript levels. The fold induction of Smad7 is calculated relative to DMSO vehicle treatment)

To develop a specific negative control for HF, we undertook to modify HF into a non-functional analog that was structurally similar to the parent compound, but did not elicit effects on the early transcriptional target Smad7. Previous reports studying the structure activity relationship (SAR) of the anti-malarial activity of HF and febrifugine analogs provided valuable insights for the design of active and inactive analogs as control compounds [25-28]. In general, modifications to the quinazolone-system were tolerated, while blocking the basic piperidine nitrogen did result in inactivation of the antimalarial activity (Ralph Mazitschek, unpublished results). In an effort to synthesize analogs that would be suitable for the development of an affinity purification matrix for the molecular targets of HF, we have identified MAZ1319 as suitable ligand (Figure 2). In MAZ1319, (6-chloro-3-(3-((2S/R, 3R/S)-3-hydroxypiperidin-2-yl)-2-oxopropyl)-7-((trimethylsilyl)ethynyl)quinazolin-4(3H)-on the Br on the quinazoline is substituted with a bulky trimethyl silyl (TMS) group. In contrast the acylation of the piperidine nitrogen as *tert*-butyl carbamante, MAZ1310 ((2S/R,3R/S)-tert-butyl 2-(3-(7-bromo-6-chloro-4-oxoquinazolin-3(4H)-yl)-2-oxopropyl)-3-hydroxypiperidine-1-carboxylate), was not tolerated (Figure 2).

The relative activation of Smad7 transcription by HF, MAZ1310 and MAZ1319 was determined in NMuMG cells, in which HF was previously reported to induce Smad7 expression (Figure 2) [14]. To test analog activity, NMuMG cells were treated for 8 hours with increasing concentrations of HF, MAZ1310 or MAZ1319 ranging from 0.010 μM to 1 μM, and Smad7 expression levels were analyzed by quantitative RT-PCR. A minimum of 0.03 μM HF was required to induce Smad7 transcription in this assay, while 0.10 μM MAZ1319 induced the expression of Smad7. In contrast, MAZ1310 did not activate Smad7 transcription at any concentration tested. Thus, the addition of a bulky substituent on the quinazoline, while reducing HF potency, did permit downstream transcriptional regulation. In contrast, blocking of the secondary amine on the piperidine ring in MAZ1310 caused a complete loss of HF transcriptional activity.

### HF induces Mmp13 transcription without affecting TGFβ signaling

**Figure 3:**
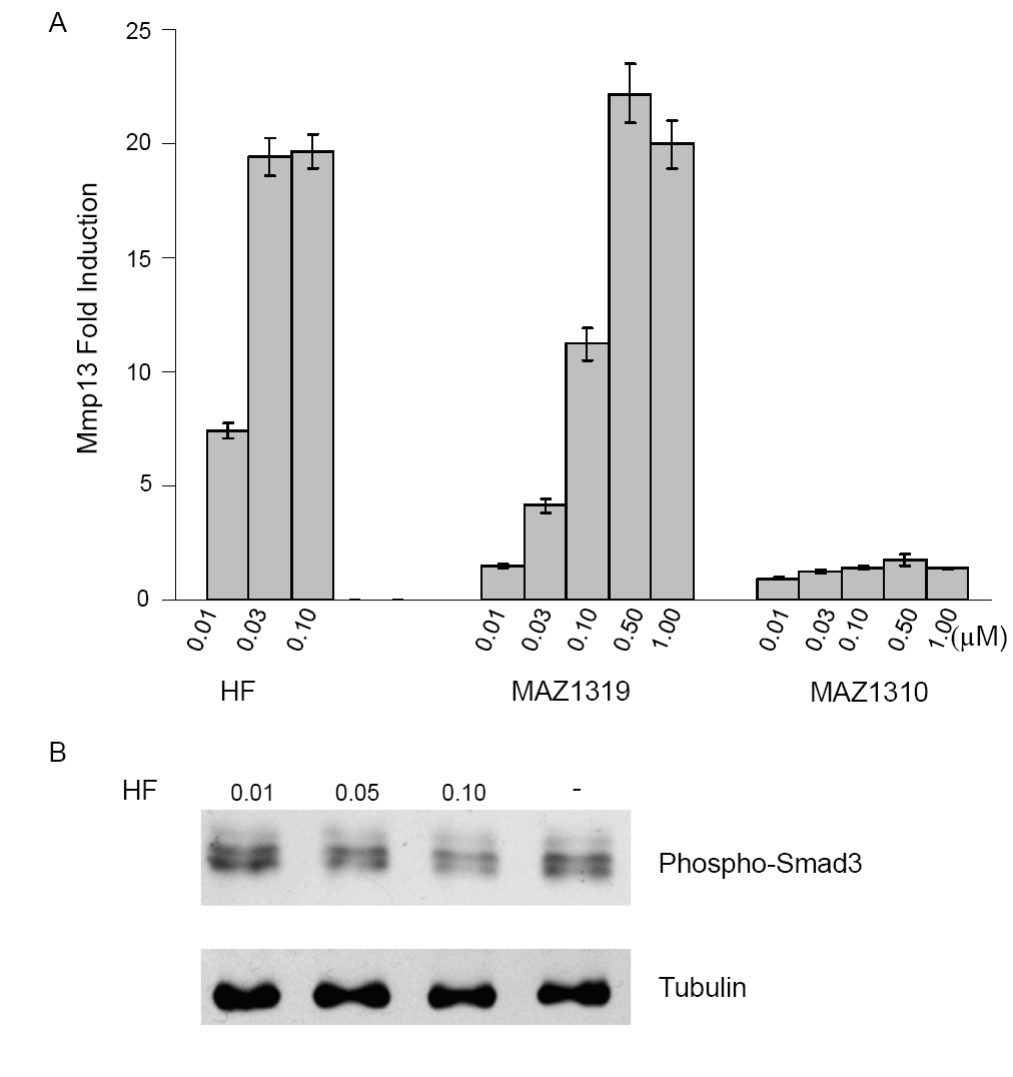
**HF induces Mmp13 expression in assay that does not inhibit TGFβ signaling or induce Smad 7**. (A) HF and MAZ1319 but not MAZ1310 induce transcription of Mmp13 (A) in NMuMG cells after 8 hours of treatment, consistent with activities determined for HF analogs for Smad7 induction. Note that 0.01 μM HF treatment induces Mmp13 expression, but not Smad7 expression in this assay. All samples were normalized to β-Actin transcript levels. The fold induction of Mmp13 is calculated relative to DMSO vehicle treatment. (B) HF does not affect endogenous Phospho-Smad3 levels in cells at minimum concentration required to induce Mmp13 expression (0.01 μM). Cells were treated with the indicated HF concentrations or with DMSO (-) and were cultured in complete media for 8 hours. Cells were harvested after 8 hours and processed for SDS-PAGE analysis. Phospho-Smad3 was detected using a polyclonal Phospho-Smad3 antibody and Tubulin levels are shown as a loading control.

To assay the biological activity of the HF analogs more broadly, we determined the effects of HF derivatives on Mmp13 expression. Mmp13 was previously reported to be up-regulated by HF in hepatic stellate cells [22]. Consistent with the relative effects of the HF analogs on Smad7 expression, MAZ1319, but not MAZ1310 activated Mmp13 transcription (Figure 3). However, Mmp13 expression was induced by as little as 0.01 μM HF, a three fold lower concentration than the minimum required to induce Smad7 transcription. MAZ1319 also exhibited approximately 3 fold higher potency in activating Mmp13 transcription relative to Smad7 activation. These results are intriguing in light of the critical role that has been proposed for Smad7 in HF biological activity [14].

Since Smad7 is an inhibitor of TGFβ signaling, we tested whether or not 0.01 μM HF treatment results in TGFβ inhibition and found no detectable decrease in activated Smad3 under these (Figure 3). Reduction of phosphorylated Smad3 levels required treatment with a substantially higher dose of HF (0.1 μM), consistent with previously published reports [14]. Since Mmp13 induction occurs at a dose of HF at which neither induction of the TGFβ inhibitor Smad7 nor a decrease in the level of TGFβ signaling in these cells can be observed, it seems unlikely that Mmp13 induction is mediated through effects on TGFβ signaling. To explore the molecular basis for the observed transcriptional activity, we generated a global expression profile for HF under conditions where Mmp13 but not Smad 7 expression is altered by HF.

### Gene expression profiling reveals HF activates expression of Integrated Stress Response effectors

A broad spectrum HF expression profile was generated by microarray transcriptional analysis. NMuMG cells were treated with 0.01 μM HF or 0.01 μM MAZ1310 for 8 hr. Each treatment was done in triplicate. Cells were then harvested and total RNA was extracted. RNA samples were processed for microarray analysis using the Mouse One Array gene chip. All arrays were performed in triplicate. Microarray data is available via the Gene Expression Omnibus, record number GSE30667. As expected, Mmp13 expression was up-regulated, but not Smad7.

**Figure 4:**
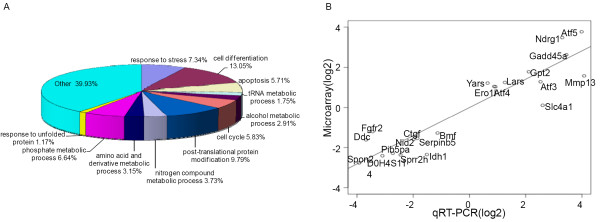
**Global expression profiling implicates multiple biological pathways downstream of HF**. (A) Relative abundance of HF transcriptional targets based on biological ontogeny. Biological processes which accounted for one percent or more and p < 0.001 are specifically shown. Note that stress response and unfolded protein response characteristic genes are among those showing enrichment. Enrichment analysis was performed using The Database for Annotation, Visualization and Integrated Discovery (**DAVID**) v6.7. (B) Validation plot showing high correlation between array results and validation by quantitative RT-PCR. Fold-change values of 21 genes from the microarray (y-axis) are plotted against the corresponding values from the qRT-PCR (x-axis). Each value is the log2-ratio of the treated vs. the untreated samples. The slope of the regression line is 0.704 and the Pearson correlation coefficient is 0.938 (p-value 1.33e-11), indicating highly significant agreement.

**Figure 5:**
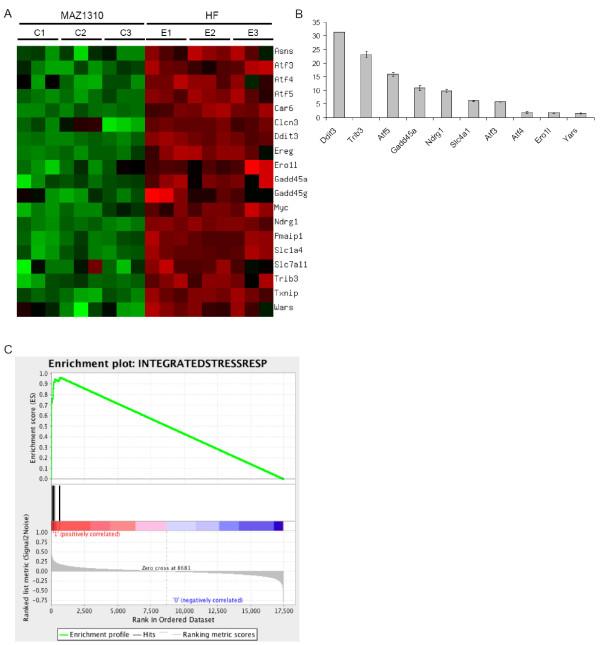
**HF upregulates the expression of Integrated Stress Response effector genes**. (A) A heat-map of signature genes related to the Integrated Stress Response (ISR) is displayed. Among reported genes in previous studies, nineteen genes were chosen if absolute fold-change was greater than 2 and q-value was less than 0.001. Measurement values were normalized to have mean zero and variance one per gene. The heat-map was generated using matrix2png. (B) Validation of HF induction of selected ISR genes by quantitative RT-PCR (qRT-PCR). qRT-PCR was preformed on an independent set of samples from an 8 hour 0.01 μM HF or MAZ1310 treatment. qRT-PCR data is expressed as the mean fold change in the level of the indicated transcript between HF and MAZ1310 treated samples, and the standard error of the means (SEM) is included. Each condition was assayed in triplicate, and the experiment was repeated three times. Aggregate results are shown. (C) GSEA plot. Enrichment Score (ES) was 0.962 and its nominal P-value < 10^-3^. **top**: Enrichment profile where running ES for the Atf4/ISR gene set as the analysis walks along the ranked list; **middle**: The Hits shows where the members of the Atf4/ISR gene set appear in the ranked list; **bottom**: Ranked list metric measures a gene's correlation with a phenotype (i.e. Halofuginone treatment).

HF treatment altered the expression of multiple genes, involved in a variety of biological processes including signal transduction, transcriptional regulation, redox and cell metabolic homeostasis demonstrating that HF exerts widespread transcriptional effects (Figure 4). Strikingly, HF potently induced the expression of signature Atf4/Integrated Stress Response effector genes including Atf5, Ddit3, Trib3, Ndrg1, Gadd45α and Slc1a4 (Figure 5) [16,23,24]. To a lesser extent, HF also induced the expression of the transcription factors Atf3 and Atf4 itself (Figure 5). When Atf4/ISR genes were tested against gene expression in HF and MAZ1310 (gene set: Atf4/ISR genes, phenotype: HF treatment), GSEA indicated a high statistical significance (Enrichment Score = 0.962, p = 0.000, Normalized Enrichment Score (NES) = 1.27 and FDR q-value = 0.000; Figure 5). All 19 Atf4/ISR genes were the core members of the *leading-edge subset *in GSEA term, meaning all genes were highly correlated with HF intervention (Table 1) and appeared before the point where the running sum reached its maximum deviation from zero (Figure 5). The up-regulation of these transcripts is characteristic of a coordinated cellular response to a set of defined stressful conditions, as mediated by the transcription factor Atf4 [16,22,23,29]. To provide an additional, independent validation of the microarray results, quantitative RT-PCR (qRT-PCR) was used to determine the relative expression of Atf5, Trib3, Ddit 3, Ndrg1, Gadd45α, and Scl1a4, Atf3, Atf4, Ero1l and Yars and consistent with the microarray results, HF, but not MAZ1310, consistently up-regulated the expression of these ISR genes (Figure 5). Activation of the Atf4/ISR in the absence of detectable TGFβ signaling inhibition in HF treated epithelial cells points to additional signaling mechanisms that may be responsible for HF effects *in vivo*.

**Table 1:** GSEA results of Atf4/ISR target genes

	PROBE	RANK IN GENE LIST	RANK METRIC SCORE	RUNNING ES	CORE ENRICHMENT
1	Ddit3	1	0.860	0.098	Yes

2	Atf5	2	0.756	0.183	Yes

3	Ndrg1	3	0.744	0.268	Yes

4	Trib3	5	0.731	0.350	Yes

5	Car6	8	0.678	0.427	Yes

6	Gadd45a	9	0.636	0.499	Yes

7	Pmaip1	13	0.562	0.563	Yes

8	Ereg	18	0.498	0.619	Yes

9	Slc1a4	20	0.491	0.675	Yes

10	Txnip	26	0.449	0.725	Yes

11	Atf3	80	0.349	0.762	Yes

12	Ero1l	124	0.317	0.795	Yes

13	Slc7a11	155	0.293	0.827	Yes

14	Wars	156	0.292	0.860	Yes

15	Clcn3	176	0.282	0.891	Yes

16	Myc	200	0.268	0.920	Yes

17	Gadd45g	262	0.248	0.944	Yes

18	Asns	593	0.189	0.947	Yes

19	Atf4	673	0.179	0.962	Yes

## Discussion

### Derivitization of Halofuginone implicates structural features required for transcriptional regulation

We have derivatized HF into functional and non-functional derivatives. MAZ1310, in which the secondary amine is blocked as carbamate, failed to induce transcriptional responses by all HF targets tested. Our data indicate that the piperidine ring is critical for HF activity, which is consistent with observations that modifications of the basic secondary nitrogen abolishes HF and febrifugine anti-malarial activity [25-28]. The basis for loss of activity in HF derivative MAZ1310 may be an inability to bind HF targets or may be due to the formation of a non-functional complex between this analog and HF binding targets. Consistent with this idea, we have found that HF, but not MAZ1310, is able to compete a recently identified HF binding protein in an in vitro affinity purification assay [30].

Conservation of functional moieties was also observed for the quinazoline ring. Addition to the quinazoline ring in MAZ1319 did not inhibit transcriptional regulation activity. However, we found that while functional, MAZ1319 was slightly less potent than its parent compound. Thus while, this compound is likely to interact with direct HF mediators, it may do so with decreased affinity, leading to reduced potency. The ability of MAZ1319 to recapitulate HF transcriptional regulation has potential implications for the development of an HF affinity reagent, which can be used for the identification of HF interacting proteins. Collectively, our work with HF derivatives suggests that the two reported functions of HF, as an anti-protozoal and as a transcriptional regulator require similarly structured binding pockets in effector proteins.

### The Atf4/Integrated Stress Response is a putative mediator of HF biological activity

HF therapeutic effects have been linked to HF-mediated inhibition of TGFβ signaling, in part through upregulation of Smad7 [7,14,15]. In contrast, the work of Sundrud and colleagues has implicated the AAR, which is one of several pathways that may specifically trigger the Atf4/ISR downstream of HF modulation of T-cell differentiation [16]. These observations do not explain prior work on HF in epithelial cells where HF has been shown to alter ECM gene expression and ameliorate fibrosis. In an effort to explore the basis for HF effects on epithelial cells, we generated a transcriptional profile of HF target genes using a physiologically relevant concentration of HF. We find that Mmp13 is up-regulated by HF in epithelial cells and that this activation is clearly separable from Smad7 activation/TGFβ inhibition. Moreover, we find that under these conditions, HF alters the expression of a broad spectrum of transcripts, and specifically up-regulates the expression of a group of genes induced by the ISR, as a consequence of Atf4 up-regulation.

The ISR is a coping mechanism that allows cells experiencing metabolic, oxidative, hypoxic, or ER stress to conserve resources while adjusting gene expression to alleviate the effects of stress conditions [23,31-33]. The core ISR program of transcriptional and translational control is activated by all of these stimuli, and includes phosphorylation and consequent inactivation of eIF2α and upregulation of Atf4 translation and transcription leading to a program of decreased protein translation and metabolic expenditure [23,31-33]. HF treatment of NMuMG epithelial cells let to the induction of Atf4 itself, as well as that of the bZIP transcription factors Atf3, Atf5 and Ddit 3. These proteins dimerize with each other and with other C/EBP family transcriptions factors and regulate the expression of other ISR effectors [29]. While Atf3 and Atf5 have contextually regulated roles as pro-survival and pro-apoptotic mediators of the ISR, Ddit3 appears to be exclusively pro-apoptotic [32,34-36]. Trib3, which was also induced by HF, is a negative feedback regulator of the Atf4-ISR, and is a transcriptional target of Ddit3 [36,37]. Gadd45α is induced by Atf4, and is a cell cycle checkpoint regulator required for proper S-phase control and for maintenance of genome stability under stressful conditions [38-40]. Consistent with a response to remediate stress, HF treatment resulted in the increased expression of multiple genes involved in amino acid import and synthesis. Specifically, elevated transcription of the amino acid transporters Slc1a4 and Slc7a11, tRNA synthetases, and asparagine synthetase (Asns) was apparent [23,24]. It is interesting to note that Asns, Atf3, Trib3 and Slc7a11 all contain amino acid response elements. Previously published expression profiles of cells undergoing nutrient deprivation, also show the same subset of genes being up-regulated, suggesting that HF treatment may mimic this stimulus [16,24,31]. In contrast, a characteristic response to ER stress is the increased transcription of ER chaperones such as GRP78/BiP, which was not detected in our expression analysis [16,33,41]. This is at least in part because the ER stress pathway activates XBP1 and ATF6 as well as the eIF2α/ATF4 cascade [16,33]. In contrast, the AAR, which is activated by unloaded tRNAs is characterized by the upregulation of amino acid transporters and genes containing amino acid response elements as seen in our array [16,24].

Work by Sundrud and colleagues showed that the effects of HF on T-cells could be completely reproduced by amino acid depletion and amino acid supplementation completely reversed the effects of HF in T-cells [16]. In light of these data and the similarity in upregulated genes previously reported to be induced by nutrient deprivation, it seems plausible that the AAR is at least in part responsible for HF effects in epithelial cells, but not necessarily restricted to this. In agreement with this idea, recent work in our laboratory has revealed that treatment with HF induces the phosphorylation of eIF2α in NMuMG cells consistent with observations on HF activation of the AAR in T cells [Malcolm Whitman unpublished data, 16]. Together, these data suggest that activation of the Atf4-ISR may be a common feature of HF treated cells in vivo and in vitro. Moreover, as in T-cells, this HF activity is clearly distinct from HF-mediated suppression of TGFβ signaling.

Since HF has been implicated in the suppression of tumor growth, metastasis, and inhibition of EMT, it is interesting to speculate on the potential role of ISR activation in mediating these effects. However, to date, these HF biological activities have largely been attributed to HF-mediated inhibition of TGFβ signaling [7,14,15]. The ability of HF to activate the ISR in multiple cell types without affecting TGFβ signaling levels presents the possibility that the Atf4/ISR should be examined as a separable mediator of HF biological effects. Our data correlate HF activation of the Atf4/ISR with changes in the transcription of multiple genes, including ECM regulators such as Mmp13 which was previously linked to HF anti-fibrotic effects on fibrotic potential in a liver cirrhosis model [22]. Whether this is a correlative or causal effect is the basis of current experiments in our laboratory. It has already been reported that HF activation of the Atf4/ISR via AAR stimulation is sufficient to explain HF biological effects in T-cells, and in turn HF-dependent amelioration of autoimmune disease [16]. At present, the mechanism by which Atf4/ISR activation and or the AAR specifically could modulate fibrotic disease is not known. It is interesting, however, that the Atf4/ISR was recently implicated as a common mechanism employed by a number of promising chemotherapeutic drugs. For example, induction of the ISR is critical for the ability of the proteosome inhibitor class of anti-tumor compounds to inhibit cell proliferation and sensitize cells to apoptosis [32,42]. Importantly, HF effects on autoimmune disease models are cytoprotective, suggesting that enhancement of apoptosis or decrease proliferation are not likely to be the molecular mechanism employed by HF in this case [16]. Current work in our laboratory is focused on examining the extent to which activation of the Atf4/ISR and the AAR specifically can mimic HF biological effects, and exploring the molecular basis for Atf4/ISR mediation of HF activity in these contexts.

## Conclusions

Our global expression profile of early, low dose HF responses indicates that activation of the ISR is a potent response to HF treatment and may be functionally relevant to HF biological effects in a multiple cell types.

## Methods

### Synthesis of HF analogs

1 kg of 10% pure HF was received as a gift from Hangpoon Chemical Co. (Seoul, South Korea), which was further purified via HPLC to >99% purity and used for experiments.

MAZ1310 (2S/R,3R/S)-tert-butyl2-(3-(7-bromo-6-chloro-4-oxoquinazolin-3(4H)-yl)-2-oxopropyl)-3-hydroxypiperidine-1-carboxylate. 982 mg di-tert-butyldicarbonate in 10 mL DMF were added to a solution of 1.5 g Halofuginone hydrobromide and 1.3 mL diisopropylethylamine in 100 mL. The reaction mixture was stirred for 16 h at room temperature. After addition of water the aqueous layer was extracted three times with diethyl ether. The combined organic layers were dried over sodium sulfate and evaporated to dryness. The crude product was purified on silica gel with dichloromethane/methanol to yield the desired Boc protected product as white solid in quantitative yield.

MAZ1319 (6-chloro-3-(3-((2S/R,3R/S)-3-hydroxypiperidin-2-yl)-2-oxopropyl)-7-((trimethylsilyl)ethynyl)quinazolin-4(3H)-one). 10 mg of (2S/R,3R/S)-tert-butyl 2-(3-(6-chloro-4-oxo-7-((trimethylsilyl)ethynyl)quinazolin-3(4H)-yl)-2-oxopropyl)-3-hydroxypiperidine-1-carboxylate are dissolved in 500 uL dichloromethane, followed by the addition of 50 uL TFA. The reaction mixture is stirred at room temperature for 5 h and the solvent is removed under reduced pressure. The product is used without further purification as trifluoroacetate.

### NMuMG cell culture and treatment with HF

NMuMG cells were a gift from Dr. Sheila Thomas (Harvard Medical School/Beth Israel Deaconess Medical Center) and were cultured in DMEM supplemented with 10% FBS, 10 ug/ml Insulin (Sigma Aldrich) and Pen/Strep. For treatment of NMuMG cells, cells were freshly plated into complete growth media and 45 minutes later HF or HF analogs diluted in acidified DMSO was added at the indicated concentrations.

### Immunohistochemistry

NMuMG cells were treated with the indicated HF concentrations or with DMSO (-) and were cultured in complete media for 8 hrs prior to harvest. To assess Phospho-Smad3 levels, cells were lysed in modified RIPA (50 mM Tris, 150 mM NaCl, 25 mM β-glycerophosphate, 100 mM sodium fluoride, 2 mM sodium orthovanadate, 10 mM sodium pyrophosphate, 20 nM Calyculin, cOmplete EDTA-free protease inhibitors mix (Roche Applied Science), 3 mM PMSF, 1 mM EDTA, 1% NP-40, 0.5% sodium deoxycholate, and 1 mM DTT). Phospho-Smad3 was detected with Phospho-Smad3 (Ser423/425)/Smad1 (Ser463/465) antibody (Cell Signaling).processed by SDS-PAGE analysis. 2 ng/ml TGFβ stimulation of NMuMGs for 30 minutes in this assay was used as a positive control to determine size of Phospho-Smad3 on the Western blot. Monoclonal anti-α-Tubulin Clone DM1A (Sigma Aldrich).

### Quantitative RT-PCR

NMuMG cells were harvested 8 hr post treatment with varying concentrations of HF, vehicle or derivatives MAZ1319 and MAZ1310 and washed with ice-cold PBS. Samples for each PCR experiment were in biological triplicate. Total RNA was extracted using TRIzol Reagent (Invitrogen) according to the manufacturer's protocol. The extracted RNA was then treated with RQ1-DNAse I (Promega) for 30 min and subsequently extracted with Phenol-Chloroform-Isoamyl Alcohol (Ambion), followed by a Chloroform (Sigma) extraction and precipitated with Ammonium Acetate/Ethanol. Total RNA was quantitated and checked for purity on the NanoDrop1000 spectrophotometer (Thermo Scientific). 0.5 μg total RNA was used as template per cDNA reaction. cDNA reactions were carried out according to standard protocols using oligo-dT (Roche Applied Science) primer, MMLV-RT (Promega) reverse transcriptase and RNAse Out (Invitrogen). A no RT control was included for each experiment. Quantitative RT-PCR (qRT-PCR) was performed on the LightCycler480 (Roche) using Universal Probe Library (Roche) probe-based PCR method. RT-PCR primers for each target were designed using to Roche Universal Probe Library Assay Design Center (https://www.roche-applied-science.com/sis/rtpcr/upl/acenter.jsp?id=030000) with sequences and appropriate UPL probes. RT-PCR reactions were performed according the manufacturers protocol using the Universal Probe Library LightCycler480 Probes Master (Roche) reaction mix. qRT-PCR conditions were as follows: 1 Cycle: 95°C 10 min; 45 Cycles: 95°C 10 sec, 60°C 30 sec, 72°C 1 sec; 1 Cycle: 40°C 30 min. Ct values were calculated using the Roche Light Cycler 480 Software Release 1.2.0.0625 Analysis Program. Each PCR was done in triplicate. All samples were normalized to Beta Actin and the relative concentration of each sample was calculated using the ddCt method relative to DMSO or MAZ1310 treated samples as indicated. Each experiment was repeated at least in duplicate. The fold change in expression of each target gene was calculated using the mean relative concentrations from each experiment as compared to DMSO vehicle or MAZ1310 treatments alone, as indicated. p values were calculated using the Student's T-test.

### Microarray analysis

NMuMG cells treated with 10 nM HF or 10 nM MAZ1310 for 8 hrs in complete media. Each treatment was done in biological triplicate. Cells were then harvested and total RNA was extracted as described previously. Purified RNA samples were submitted to Phalanx Biotech for microarray analysis. A detailed description of Phalanx Biotech company microarray procedures can be found at http://www.phalanxbiotech.com/. We used Phalanx Mouse Whole Genome OneArray to measure mRNA abundance of HF-treated and MAZ1310 control samples. Each array was performed in triplicate. A probe was excluded from the data analysis if it had negative/missing measurement value in any of eighteen samples. The resulting 21,973 probes were used to perform quantile-normalization to have the same distribution of measurement values across the eighteen samples [43]. Then 21,973 probes were mapped into the official gene symbols and were aggregated into 17,454 genes by taking median value of the multiple measurements for each gene. A moderated t-test was used to detect differentially expressed genes between HF treated and MAZ1310 control samples [44]. Q-value was used as a measure of significance to take into account simultaneous tests of thousands of genes [45,46]. We conducted Gene Set Enrichment Analysis (GSEA), a computational method that determines whether *a priori *defined set of genes show significantly significant, concordant differences between two phenotype classes [47]. In our case, 19 genes Atf4/ISR genes constitute as a predefined set and HF status is a phenotype with two classes, HF and MAZ1310.

## Abbreviations

HF: Halofuginone; ISR: Integrated Stress Response; AAR: Amino acid restriction response; ECM: extracellular matrix; EMT: epithelial to mesenchymal transition; TGFβ: Transforming Growth Factor-β.

## Competing interests

The author declares that they have no competing interests.

## Authors' contributions

YGK and MW conceived the study. YGK designed and carried out all experimental studies, except as described below and wrote the paper manuscript. JK carried out normalization, statistical analysis of microarray data and GSEA analysis. RM purified HF, designed, synthesized and purified all chemical derivatives described. WPK facilitated and coordinated interaction with Phalanx BioTech Group, provided access to the Roche UPL library for real-time PCR validation of microarray data and provided guidance and expertise for microarray data analysis. MW helped conceive of the study and participated in its design. All authors read and approved the final manuscript.
